# The complete chloroplast genome of *Vitis heyneana* Roem. et Schult., an economic plant to China

**DOI:** 10.1080/23802359.2019.1710289

**Published:** 2020-01-14

**Authors:** Binbin Qin, Yujuan Liu, ZeYun Huang, Jing Liu, Qinpei Chen, Guolun Jia, Jun Xie

**Affiliations:** a State Key Laboratory of Seeding Bioengineering, Ningxia Forestry Institute, Yinchuan, Ningxia, China;; b Ningxia Forestry Technology Popularization Station, Yinchuan, Ningxia, China

**Keywords:** Chloroplast genome, Illumina sequencing, phylogenetic analysis, *Vitis heyneana*

## Abstract

The chloroplast (cp) genome sequence of *Vitis heyneana* has been characterized from Illumina pair-end sequencing. The complete cp genome was 160,830 bp in length, containing a large single-copy region (LSC) of 89,049 bp and a small single-copy region (SSC) of 19,071 bp, which were separated by a pair of 26,355 bp inverted repeat regions (IRs). The genome contained 131 genes, including 88 protein-coding genes, 37 tRNA genes, and 8 rRNA genes. The overall GC content is 37.4%, while the corresponding values of the LSC, SSC, and IR regions are 35.3, 31.7, and 43.0%, respectively. Further, the phylogenetic analysis suggested that *V. heyneana* was closely related to *Vitis ficifolia*.

*Vitis heyneana* Roem. et Schult., is one of striking wild germplasms of the East Asian *Vitis* spp. It often grows in hillside or ravine on hillsides at elevations of 100 m to 3200 m in the west of China such as Shanxi, Shaanxi, Gansu, Sichuan, Guizhou, Yunnan, and Xizang provinces of China (Chen et al. 1987). It is one of the most widely distributed wild species in east Asia. At present, grapes are mainly made available as fresh edible fruit and a small part have also been used for making wines in China. The fruit traits of *V. heyneana* have several advantages, such as high Sugar and acid. The wine made by *V. heyneana* is a quality wine rich in phenols and proanthocyanidins (Wu 2010; Lin et al. 2005). However, little is known about the genetic diversity and genetic differentiation among the populations of *V. heyneana*. Such information is essential for developing optimum conservation and management strategies for *V. heyneana*. In this study, we report the first complete plastome of *V. heyneana*, and assessed its phylogenetic position within Vitaceae.

One individual of *V. heyneana* was sampled from the Yinchuan Botanical Garden (38°28′N, 106°16′E; Ningxia, NW China). Total DNA was isolated using the DNeasy plant Mini Kit (Qiagen, Carlsbad, CA) and stored in state key laboratory of seeding bioengineering, Ningxia Forestry Institute, the number is 2009PC0926. The strategy for sequencing, assembly, and annotation of the chloroplast genome was adopted from Yao et al. (2015). The sequence of chloroplast genomes was deposited in GenBank (accession number MN398393).

The complete chloroplast genome of *V. heyneana* is 160,830 bp in length. The complete cp genome exhibit the typical quadripartite structure, including a large single-copy (LSC) region of 89,049 bp, a small single-copy (SSC) region of 19,071 bp, and two inverted repeat (IR) regions of 26,355 bp each. The cp genomes encode an identical set of 131 genes in total, including 88 protein-coding genes (PCGs), 8 rRNA genes, and 37 tRNA genes. Among all of these genes, four rRNA genes (i.e. *4.5S*, *5S*, *16S*, and *23S rRNA*), eight PCGs (i.e. *ndhB*, *rpl2*, *rpl23*, *rps7*, *rps12*, *ycf1*, *ycf2*, and *ycf15*), and seven tRNA genes (i.e. *trnA-UGC*, *trnI-CAU*, *trnI-GAU*, *trnLCAA*, *trnN-GUU*, *trnR-ACG*, and *trnV-GAC*) occur in double copies. The overall GC-content of *V. heyneana* cp genome is 37.4%, while the corresponding values of the LSC, SSC, and IR regions are 35.3, 31.7, and 43.0%, respectively.

To further understand the chloroplast genome of *V. heyneana* and study the evolution of genus *Vitis*, the concatenated nucleotide sequences of 18 PCGs from 37 *Vitis* species and three outgroup species from Ampelopsis were used for the phylogenetic analysis. Their complete mitochondrial sequences were obtained from GenBank of NCBI. Bayesian analyses were performed with MrBayes 3.1.2 (Ronquist and Huelsenbeck 2003), and the BI phylogenetic tree showed that *V. heyneana* is closely related to *Vitis ficifolia* (Figure 1).

**Figure 1.**
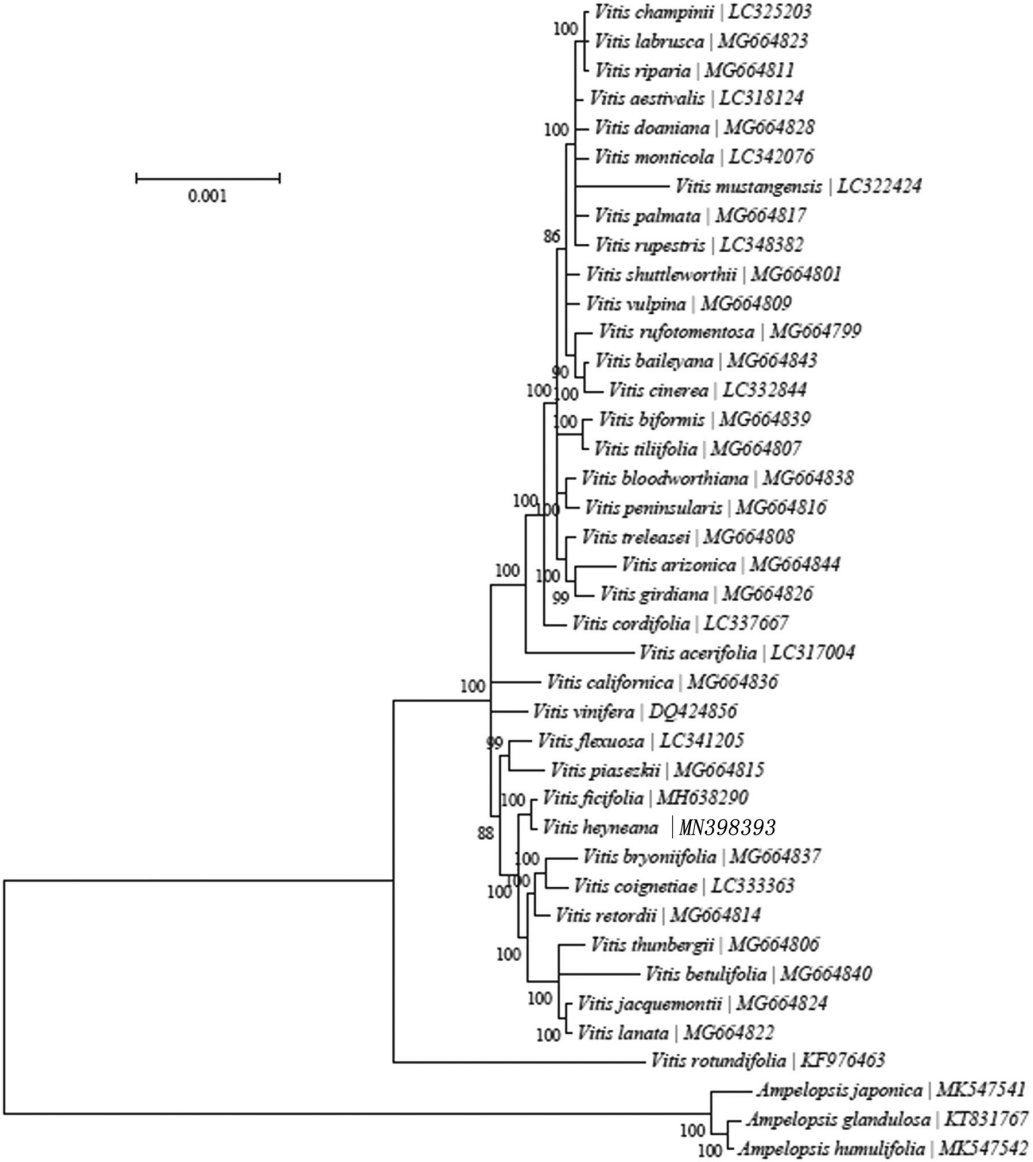
The BI phylogenetic tree of *V. heyneana* based on the concatenated nucleotide sequences of 18 PCGs. Bayesian posterior probabilities values are given above the branches.
